# Induced DNA demethylation by targeting Ten-Eleven Translocation 2 to the human *ICAM-1* promoter

**DOI:** 10.1093/nar/gkt1019

**Published:** 2013-11-03

**Authors:** Hui Chen, Hinke G Kazemier, Marloes L. de Groote, Marcel H. J. Ruiters, Guo-Liang Xu, Marianne G. Rots

**Affiliations:** ^1^Epigenetic Editing, Department of Pathology and Medical Biology, University Medical Center Groningen, University of Groningen, Hanzeplein1, 9713 GZ Groningen, The Netherlands, ^2^The State Key Laboratory of Molecular Biology, Institute of Biochemistry and Cell Biology, Chinese Academy of Sciences, 320 Yueyang Road, Shanghai 200031, China and ^3^Synvolux Therapeutics Inc., LJ. Zielstraweg 1, 9713 GX Groningen, The Netherlands

## Abstract

Increasing evidence indicates that active DNA demethylation is involved in several processes in mammals, resulting in developmental stage-specificity and cell lineage-specificity. The recently discovered Ten-Eleven Translocation (TET) dioxygenases are accepted to be involved in DNA demethylation by initiating 5-mC oxidation. Aberrant DNA methylation profiles are associated with many diseases. For example in cancer, hypermethylation results in silencing of tumor suppressor genes. Such silenced genes can be re-expressed by epigenetic drugs, but this approach has genome-wide effects. In this study, fusions of designer DNA binding domains to TET dioxygenase family members (TET1, -2 or -3) were engineered to target epigenetically silenced genes (*ICAM-1, EpCAM*). The effects on targeted CpGs’ methylation and on expression levels of the target genes were assessed. The results indicated demethylation of targeted CpG sites in both promoters for targeted TET2 and to a lesser extent for TET1, but not for TET3. Interestingly, we observed re-activation of transcription of *ICAM-1*. Thus, our work suggests that we provided a mechanism to induce targeted DNA demethylation, which facilitates re-activation of expression of the target genes. Furthermore, this Epigenetic Editing approach is a powerful tool to investigate functions of epigenetic writers and erasers and to elucidate consequences of epigenetic marks.

## INTRODUCTION

Epigenetics is the study of heritable changes of gene expression regulation without a change in the DNA base sequence. Epigenetic marks, including DNA methylation to form 5-methylcytosine (5-mC) and histone modifications, play an important role in, e.g. X chromosome inactivation, retrotransposon silencing, genomic imprinting and maintenance of epigenetic memory (1–4). Although it was originally thought to be a stable epigenetic characteristic, cytosine methylation is a dynamic and reversible process (5). 5-mC demethylation occurs in many physiological processes, such as zygotic epigenetic reprogramming, early embryonic development, somatic cell reprogramming, removal of gene imprinting and developing primordial germ cells (6–13). In addition, genome-wide analysis of DNA methylation patterns in pluripotent and differentiated cells at single-nucleotide resolution indicated that DNA methylation can be dynamically regulated during cellular differentiation (14,15). These observations suggest the existence of a mammalian enzymatic activity, capable of erasing or modifying pre-existing DNA methylation patterns. However, the mechanisms of active DNA demethylation are still poorly understood (16).

Recently, 5-hydroxymethylcytosine (5-hmC) was discovered as a new epigenetic mark, and suggested to be an intermediate in the process of active DNA demethylation (17). 5-hmC is the product of 5-mC hydroxylation, and was first discovered in phage DNA in 1952 (18). Later, 5-hmC was found in the brain of *Rattus norvegicus*, *Mus musculus* and *Rana catesbiana* (19), although subsequent studies have failed to reproduce these results (20). Recently, 5-hmC was reported to exist in the vertebrate brain and in several other tissues (21–24). Interestingly, although 5-hmC exists in mouse embryonic stem (ES) cells at high levels, it decreases significantly after ES cell differentiation (17,25), to rise again in terminally differentiated cells, such as Purkinje neurons (22), which suggests a significant biological role for 5-hmC in mammalian development.

In addition to this, the Ten-Eleven Translocation (TET1, -2 or -3) family was identified as 5-mC dioxygenases responsible for catalyzing the conversion from 5-mC to 5-hmC, a process dependent on 2-oxoglutarate and iron (II) (17,26,27). The discovery of TET proteins and their biological function provides new insights in 5-mC demethylation mechanisms and points to 5-hmC as an important intermediate in the 5-mC demethylation process. Recent studies suggest that there might be multiple pathways or mechanisms by which 5-hmC and TET proteins regulate DNA methylation dynamics and gene transcription. A possible mechanism involves 5-hmC deamination by activation-induced deaminase to generate 5-hydroxymethyluracil, which can then be recognized and excised to generate an abasic site by thymine-DNA glycosylase (TDG) (28). The lesion is repaired through the incorporation of an unmethylated C by the base excision repair machinery (Supplementary Figure S1) (29). In addition, TET proteins can also further oxidize 5-hmC to 5-formylcytosine and 5-carboxylcytosine, which can subsequently be recognized and excised by TDG *in vitro* and *in vivo*, again resulting in incorporation of unmodified C by the base excision repair machinery (Supplementary Figure S1) (21,30–32). Furthermore, a recent study showed that carboxy cytosine may also be directly decarboxylated by an unknown decarboxylase present in mouse ES cells (Supplementary Figure S1) (33). Taken together, these studies suggest that the initial oxidation of 5-mC to 5-hmC by the TET family is a prerequisite for the subsequent demethylation processes, regardless of how the final steps are mediated, to complete the process of DNA demethylation (30,31,34).

Because many diseases are associated with aberrant DNA hypermethylation profiles (35–37), the removal of methylation marks to modulate gene expression in a gene-targeted way (Epigenetic Editing) would offer a novel approach in biomedical research to develop targeted epigenetic interventions (38). Although enzymes removing certain histone methylation marks have been well identified in the past decade (39), true DNA demethylation enzymes are currently unknown. In this study, we set out to demonstrate that TET proteins function as effective DNA demethylation inducers. Towards this aim, TET enzymes were fused to two different DNA binding zinc fingers (ZFs), designed to bind an 18-bp sequence in the promoters of either the InterCellular Adhesion Molecule-1 (*ICAM-1*) or Epithelial Cell Adhesion Molecule (*EpCAM*). Previously, we demonstrated that these epigenetically silenced model genes could be re-expressed from their genomic loci by targeting a transient activation domain VP64 fused to these ZFs (40,41). In the current article, the *ICAM-1*- and *EpCAM*-targeting ZFs were fused to the catalytic domains (CDs) of TET1, -2 or -3 to evaluate their ability to induce TET-mediated DNA oxidative demethylation. On one hand, this targeting strategy provides the possibility to further study the molecular mechanisms in the DNA demethylation process. On the other hand, we provide a mechanism to induce targeted gene demethylation, which together with other editing approaches of histone marks might result in re-activation or upregulation of expression of the target genes.

## MATERIALS AND METHODS

### Plasmid construction

Mouse TET1CD, -2CD and -3CD were amplified from plasmid pcDNA3-Flag-TET1CD, -TET2CD or -TET3CD (31) with Phusion Hot Start II High-Fidelity DNA Polymerase (Thermo Scientific, Leon-Rot, Germany) using forward and reverse primers introducing MluI and PacI restriction sites at the 5′ and 3′ end, respectively. These amplification products were inserted into pMX-ZF_B_-VP64-IRES-GFP [encoding the ZF recognizing the *EpCAM* promoter fused to a tetramer of Herpes Simplex Virus Viral Protein (VP) 16 (VP64)] (41) modified to include a MluI site. Using restriction enzymes MluI (Thermo Scientific) and PacI (New England Biolabs), the amplification product was inserted downstream of the ZFP by sticky-end ligation with T4 ligase (Thermo Scientific). To obtain pMX-CD54-IRES-GFP fusion constructs, the ZF_B_ was replaced with the CD54 (originally named CD54-opt31) ZF (recognizing the *ICAM-1* promoter; kindly provided by C.F. Barbas III, the Scripps Institute, La Jolla, CA, USA) (42) using the SfiI restriction enzyme. The enzymatically inactive pMX-CD54-TET1CD mutant and pMX-CD54-TET2CD mutant were obtained with site-directed mutagenesis on wild-type pMX-CD54-TET1CD and pMX-CD54-TET2CD, respectively (Supplementary Figure S2). Each zinc finger-effector domain (ZF-ED) construct contains a nuclear localization signal and a terminal hemagglutinin (HA) decapeptide tag. We verified all polymerase chain reaction (PCR)-cloned constructs by DNA Sanger sequencing (Baseclear, Leiden, The Netherlands).

### Cell culture

The packaging cell line human embryonic kidney HEK293T and human ovarian cancer cell line A2780 were cultured in Dulbecco’s Modified Eagle’s Medium (BioWhittaker, Walkersville, MD, USA) supplemented with 10% fetal bovine serum, 2 mM l-glutamine and 50 μg/ml gentamicin sulfate. Cells were cultured at 37°C in a humidified 5% carbon dioxide -containing atmosphere.

### Retroviral transductions

HEK293T cells were transfected with retroviral vector pMX-IRES-GFP encoding the ZF-ED, together with the accessory plasmid pMDLg/pRRE and packaging plasmid pMD2.G using a standard calcium-phosphate protocol to produce retroviral particles (as described previously) (43). As a control, parallel transfections were performed with the empty pMX plasmid. Host cells A2780 were seeded with a density of 2 × 10^5^ cells in T25 flasks or 6.75 × 10^5^ cells in T75 flasks. Forty-eight and 72 h after transfection, viral supernatants were supplemented with fetal bovine serum and 5 µg/ml polybrene (Sigma, St Louis, MO, USA) and used to transduce the A2780 cells, with the respective ZF-ED constructs, ZF only and empty vector. Seventy-two h after the last transduction, cells were harvested for sorting of GFP-positive cells, analysis of ZF-ED protein expression, genomic DNA extraction for subsequent pyrosequencing and total RNA extraction.

### Detection of ZF-ED fusion protein expression by immunoprecipitation and western blot

For the immunoprecipitation assay, one T25 flask of infected A2780 cells was lysed in RIPA buffer (25 mM Tris-HCl pH 7.6, 150 mM NaCl, 1% NP-40, 1% sodium deoxycholate, 0.1% sodium dodecyl sulfate, Thermo Scientific), microcentrifuged for 10 min at 4°C (14000×g) and the supernatant was transferred to a new tube. Then, 0.75-mg protein A magnetic beads (Life, Bleiswijk, The Netherlands) were incubated with rabbit polyclonal anti-HA tag antibody (Novus Biologicals, Cambridge, UK) at room temperature for 30 min. Supernatants were added and rotated at 4°C overnight. Immunoprecipitates were collected and washed four times with RIPA buffer. Proteins in the immunoprecipitates were analysed by standard western blotting. After blotting, the membranes were blocked 1 h with 5% dried milk in TBS supplemented with 0.1% Tween-20 (TBST). Then, the blot was incubated with mouse monoclonal anti-HA tag antibody (Covance, Rotterdam, The Netherlands) at 4°C overnight, followed by detection with horseradish peroxidase-conjugated secondary rabbit anti-mouse and swine anti-rabbit antibodies (Dako, Glostrup, Denmark). Visualization was done using the Pierce ECL2 chemoluminescence detection kit (Thermo Scientific, Rockford, USA).

### Detection of transduction efficiency by flow cytometry

To evaluate the transduction efficiency of A2780 ovarian cancer cells by pMX-ZF-ED-IRES-GFP constructs, fluorescence-activated cell sorting (FACS) analysis for GFP expression was performed. A2780 cells were harvested 72 h after transduction, washed three times with cold phosphate buffered saline, resuspended in phosphate buffered saline and GFP expression was analyzed using a BD FACSCalibur flow cytometer (Becton Dickinson Biosciences, San Jose, CA, USA).

### Target gene mRNA expression by quantitative real-time PCR

Total RNA from both untreated and transduced cells was extracted using the RNeasy plus mini kit (Qiagen) and 1 µg was used for subsequent cDNA synthesis with random hexamer primers using the RevertAid cDNA synthesis kit (Fermentas). *ICAM-1*, *EpCAM* and glyceraldehyde-3-phosphate dehydrogenase (*GAPDH*) expression was quantified using 10 ng cDNA, Rox enzyme mix (Thermo Scientific) and Taqman gene-specific primer/probes (*ICAM-1*: Hs00164932___m1; *EpCAM*: Hs00158980___m1, Applied Biosystems; *GAPDH*: Supplementary Table S1, Eurogentec) for 40 cycles with ABI ViiA7™ real-time PCR system (Applied Biosystems, Carlsbad, CA, USA). *GFP* expression was quantified using 10 ng cDNA, Absolute QPCR SYBR Green ROX mix (Thermo Scientific) and gene-specific primers (Supplementary Table S1) for 40 cycles with ABI ViiA7™ real-time PCR system (Applied Biosystems, Carlsbad, CA, USA). Data were analyzed with ViiA7 RUO software (Applied Biosystems) and expression levels relative to *GAPDH* were determined with the formula 2^−^^ΔCt^. Fold increase in gene-expression compared with controls was calculated with the formula 2^−^^ΔΔCt^. Samples for which no amplification could be detected were assigned a Ct value of the total number of PCR cycles.

### Methylation analysis by bisulfite sequencing and pyrosequencing

For DNA methylation analysis of the target regions, genomic DNA was extracted with Quick-gDNA™ MiniPrep kit (D3007, Zymo Research via Baseclear) and bisulfite converted using EZ DNA Methylation-Gold Kit (Zymo Research) following the manufacturer’s protocol (alternative 2). Bisulfite-converted DNA was amplified with nested PCR using specific primers. The PCR products were gel extracted using the DNA Extraction Kit (Qiagen) and cloned into pCR 2.1 vectors (TA cloning kit, Invitrogen), and individual clones were sequenced by Baseclear using M13 primers.

Five and three CpG sites in the target region of *ICAM* and *EpCAM* promoter were selected for quantitation of methylation, respectively. Bisulfite-converted DNA (10–20 ng) was amplified by PCR in a 25-μl reaction using the Pyromark PCR kit (Qiagen). Pyrosequencing was performed according to the manufacturer’s guidelines with a specific sequencing primer on the Pyromark Q24 MD pyrosequencer (Qiagen). Analysis of the percentage of methylation at each CpG was determined using Pyromark Q24 Software (Qiagen). Bisulfite-specific primers and the pyrosequencing primer information are presented in Supplementary Table S1.

### Genome-wide hydroxymethylation level detection by DNA dot-blot

Genomic DNA samples were denatured using denaturation buffer (0.4 mM NaOH, 10 mM EDTA) for 10 min at 100°C. Samples were rapidly chilled for 5 min on wet ice and then spotted on nitrocellulose membranes (BioRad, Veenendaal, The Netherlands). The membrane was baked at 80°C for 1 h and then blocked in 5% dried milk in TBST for 1 hour at room temperature. The membranes were then incubated with 1:8000 dilution of polyclonal rabbit anti-5-hmC (Active Motif, La Hulpe, Belgium) or 1:1000 dilution of monoclonal mouse anti-5-mC (Eurogentec, Maastricht, The Netherlands) overnight at 4°C. After three rounds of washing with TBST, membranes were incubated with 1:2000 dilution of horseradish peroxidase-conjugated anti-rabbit or anti-mouse IgG secondary antibody, respectively. The membranes were then washed with TBST, and visualization was done using the Pierce ECL2 Western Blotting Substrate (Thermo Scientific).

### Detection of hydroxymethylation levels at the target region by hydroxymethyl-DNA immunoprecipitation

Genomic DNA (4 μg in 450 μl TE) was sonicated to yield a fragment distribution of 300–1000 bp, and denatured by 10 min incubation at 100°C. Samples were rapidly chilled on wet ice. Then, 45 μl (10%) of denatured sample was saved as input, and the remaining sample was treated with 45 μl of 10× IP buffer (100 mM sodium phosphate at pH 7.0 [mono and dibasic], 1.4 M NaCl, 0.5% Triton X-100) and 1 μg of 5-hmC (Active Motif) or 5-mC (Eurogentec) antibody. Samples were incubated overnight at 4°C with gentle shaking. Then, 40 μl of magnetic beads (Dynabeads Protein A; Invitrogen) in 1× IP buffer was added to each sample to allow magnetic separation of the antibody bound DNA from the unbound DNA. Samples were incubated for 1 h at 4°C with rotation. Beads were collected with a magnet and washed three times with 1000 μl of 1× IP buffer for 10 min at room temperature with rotation. Beads were collected and resuspended in 250 μl of elution buffer (50 mM Tris at pH 8.0, 10 mM EDTA, 0.5% sodium dodecyl sulfate) and 10 μl proteinase K (20 mg/ml; Roche Applied Science) and incubated for 1.5 h at 50°C with constant shaking. Finally, beads were removed using the magnet. Input and sample DNA was purified using the QIAquick PCR Purification Kit (Qiagen), elution volume of 40 μl of ddH_2_O.

Subsequently, 10 ng of input or 5-hmC (or 5-mC)–enriched DNA was used in 20-μl qPCR reactions (in triplicate), each with 1× SYBR Green PCR Master Mix (ABI), 0.5 mM forward and reverse primers and water. Reactions were run on an ABI ViiA7™ real-time PCR system (Applied Biosystems) using standard cycling conditions. Fold enrichment was calculated as 2^−^^ΔCt^, where ΔCt = Ct (5-hmC enriched) − Ct (input). Primer sequences are provided in Supplementary Table S1.

### Detection of hydroxymethylation at single-base resolution for target CpG sites by combining oxidative bisulfite treatment and pyrosequencing

DNA oxidative procedure as described in the article by Booth *et al.* (44) was followed here. In summary, 800 ng of genomic DNA and 50 ng synthetic double-stranded DNA (a CpG site modified by methyl or hydroxymethyl group, respectively, Supplementary Table S2) were denatured in 0.05 M NaOH (total volume 24 μl) for 30 min at 37°C. The reaction was then snap cooled on ice for 5 min, then 1 μl of a KRuO4 (Alpha Aesar) solution (15 mM in 0.05 M NaOH) was added and the reaction was held on ice for 1 h, with occasional vortexing. The reaction was purified with a mini quick spin oligo column and followed by bisulfite treatment and pyrosequencing as described earlier.

### Statistics

All transduction experiments were performed independently for three times in triplicate. Data were analyzed using Student’s *t*-tests (one-tailed). Data were considered to be statistically significant if *P < 0.05, **P < 0.01 and ***P < 0.001. Data are expressed as mean ± S.D.

## RESULTS

### Induced hydroxymethylation in A2780 ovarian cancer cells by TET family members

Because most cultured, immortalized tumor cells display reduced 5-hmC levels (24,45,46), we first investigated whether TET dioxygenases CDs actually were able to induce hydroxymethylation in A2780 cells by ectopic overexpression of untargeted TET1, -2 or -3. All three TET dioxygenase members induced high levels of hydroxymethylation in A2780 ovarian cancer cells, as shown by DNA dot-blot (Supplementary Figure S3A). These genome-wide effects did not affect DNA methylation status or expression levels of our target genes *ICAM-1* and *EpCAM* (Supplementary Figure S3C, D and E).

### *ICAM-1*-targeted DNA demethylation

**Figure 1.**
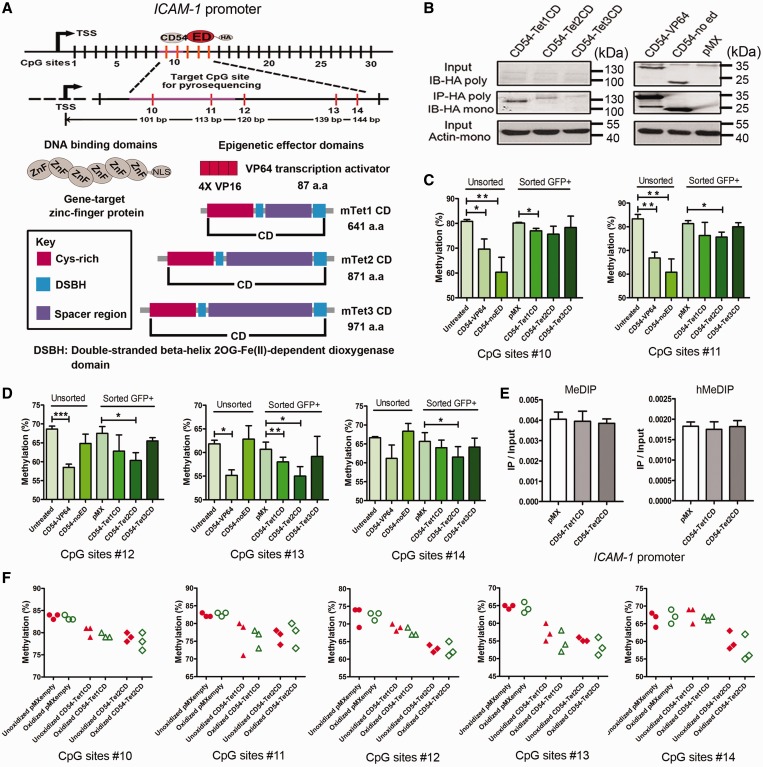
*ICAM-1*-targeted DNA demethylation. (**A**) Schematic representation of targeted DNA demethylation in *ICAM-1* promoter by epigenetic editing. The binding sites of the ZF in the promoter of *ICAM-1* are depicted, and a magnification of the target region and the actual position of each selected CpG from the transcription start site (target CpG sites are numbered #10–14, with #10 and #11 located within the ZF binding region). The purple area represents the ZFP binding site. Gray ovals represent the ZF modules, and the red ovals represent the epigenetic effector domain. A six ZFP is fused to candidate epigenetic effector domains or to the transcription activator VP64. The candidate effector domains are shown in the lower right portion of the panel: the transcription activator VP64 as the well-known positive control and the CDs of the mouse Ten-Eleven Translocation proteins (TET1, -2 and -3). Rectangular boxes display the functional domains as explained in the key box. (**B**) Protein expression of ZF fusion constructs in A2780 host cells. Upper panel: conventional western blot could only detect ZF-VP64 and ZF-only (ZF-noED); middle panel: HA-tag immunoprecipitation followed by western blot detected all ZF fusion constructs; lower panel: beta-actin was used as an input control. (**C**) Quantitative analysis of the methylation levels of target CpG sites in ZF binding region by pyrosequencing after treatment with the *ICAM-1*-targeted candidate demethylation effector domains in unsorted and sorted A2780 ovarian cancer cells. (**D**) Quantitative analysis of the methylation levels of target CpG sites in effector domain-targeted region by pyrosequencing after treatment with the *ICAM-1*-targeted candidate demethylation effector domains in unsorted and sorted A2780 ovarian cancer cells. (**E**) Examination of 5-hydroxymethylcytosine levels at *ICAM-1* promoter target region in unsorted A2780 ovarian cancer cells transduced to express CD54-TET1 or CD54-TET2CD. Quantitative PCR was performed on A2780 genomic DNA immunoprecipitated using anti-5-mC antibody (MeDIP) or anti-5-hmC antibody (hMeDIP) to evaluate the relative 5-hmC and 5-mC levels (IP/input) at the *ICAM-1* promoter. pMXempty serves as a negative control. (**F**) Quantitative sequencing analysis of methylation and hydroxymethylation levels of target CpG sites at single-base resolution by combining oxidative bisulfite treatment and pyrosequencing in sorted A2780 ovarian cancer cells transduced to express CD54-TET1 or CD54-TET2CD.

To explore the possibility of inducing targeted demethylation by TET family members, an *ICAM-1*-targeting ZF (CD54) was fused to mouse TET1, -2 or -3 CD or to the transient activation domain VP64 to obtain ZF-ED fusion proteins (Figure 1A). ZF-ED fusion protein expression was confirmed by immunoprecipitation followed by western blot (Figure 1B). Although expression levels for TET-fusion proteins were much lower than observed for CD54-VP64 or CD54-noED, bands were detected at the expected sizes, with CD54-TET1CD being more efficiently expressed than CD54-TET2CD. To determine if and which CpG sites could be demethylated in the *ICAM-1* promoter region after expression of CD54-VP64 or CD54-TET1CD, bisulfite sequencing was performed. Compared with untreated cells, cells treated to express CD54-VP64 or CD54-TET1CD demonstrate demethylation for CpG #10–14 (Supplementary Figure S4A). As no DNA demethylation was observed for CpGs located further downstream, CpG #10–14 were selected as target sites for quantitative analysis of methylation levels in single-base resolution by pyrosequencing (Figure 1).

For pMX-CD54-noED-transduced cells, a significant demethylation was observed for the ZF binding site (CpG #10: 60.0 ± 6.0%, *P* < 0.01; CpG #11: 60.8% ± 4.2%, *P* < 0.01) compared with the untreated cells (CpG #10: 80.8 ± 0.7%; CpG #11: 83.3 ± 2.2%) (Figure 1C). For pMX-CD54-VP64-transduced cells, a significant demethylation was observed for all target CpG sites (CpG #10: 69.7 ± 4.3%, *P* < 0.01; CpG #11: 66.8 ± 1.7%, *P* < 0.001; CpG #12: 58.5 ± 1.0%, *P* < 0.001; CpG #13: 55.2 ± 1.3%, *P* < 0.05; CpG #14: 61.2 ± 3.8%, *P* < 0.05) compared with the untreated cells (CpG #12: 68.7 ± 0.8%; CpG #13: 61.8 ± 0.7%; CpG #14: 66.7 ± 0.3%) (Figure 1C and D).

Although A2780 cells were readily transducible with pMX-CD54-VP64 and CD54-noED (86 ± 4% and 89 ± 3% GFP-positive cells, respectively), CD54-TET1CD, -TET2CD, -TET3CD as well as pMXempty showed a low efficiency of transgene expression (ranging from 5 ± 2% GFP-positive cells for pMXempty to 15 ± 3% GFP-positive cells for pMX-CD54-TET1CD) (Supplementary Figure S5A). To enrich for cells expressing the ZF-EDs, the cells transduced to express ZF-TET1CD,-TET2CD or -TET3CD and cells transduced with pMXempty were sorted based on GFP expression before analysis. The results showed similar *GFP* mRNA expression levels for cells transduced to express CD54-TET1CD or -TET2CD, in sorted GFP-positive as well as in unsorted cells (Supplementary Figure S5B).

For sorted pMXempty-transduced cells, no demethylation on any of the target CpG sites was observed compared with the untreated unsorted cells (Figure 1C and D). Also for sorted pMX-CD54-TET3CD-transduced cells, no significant demethylation was observed for CpG #10–14 sites compared with the pMXempty-transduced cells (Figure 1C and D). Interestingly, for sorted pMX-CD54-TET1CD-transduced cells, a significant demethylation was observed for CpG #10 and #13 sites (CpG #10: 77.0 ± 1.0%, *P* < 0.05; CpG #13: 58.0 ± 1.0%, *P* < 0.05) compared with the pMXempty-transduced cells (CpG #10: 80.2 ± 0.3%; CpG #13: 60.7 ± 1.3%) (Figure 1C and D). For sorted pMX-CD54-TET2CD-transduced cells, a significant demethylation was observed for CpG #11 (75.7 ± 2.5%, *P* < 0.05), located in the ZF binding region, and also for CpG #12 (from 67.5 ± 2.0% for pMX to 60.3 ± 1.7% for TET2, *P* < 0.05), #13 (from 60.7 ± 1.3% for pMX to 55.3 ± 2.7% for TET2, *P* < 0.05) and #14 (from 65.7 ± 1.3% for pMX to 61.5 ± 2.5% for TET2, *P* < 0.05), located in the ED target region (Figure 1C and D). To investigate whether hydroxymethylation occurs during the demethylation process on the targeted CpG sites, we used hydroxymethyl-DNA immunoprecipitation (hMeDIP) combined with real-time PCR. However, compared with cells transduced with pMXempty, we could not detect an increase in the level of hydroxymethylation in the *ICAM-1* promoter of unsorted A2780 cells after treatment with CD54-TET1CD or CD54-TET2CD (Figure 1E). As an alternative, we used pyrosequencing after oxidative bisulfite treatment to detect hydroxymethylation at single-base resolution for target CpG sites. The results indicated that compared with the unoxidized sample, there was no decrease in the level of methylation on the targeted CpG sites in sorted cells transduced to express CD54-TET1CD or -TET2CD after oxidation treatment (Figure 1F), despite the fact that we could easily detect a significant increase in hydroxymethylation of our artificially oxidized hmC control double-stranded DNA (Supplementary Figure S6B).

### Active DNA demethylation-induced *ICAM-1* gene expression

**Figure 2.**
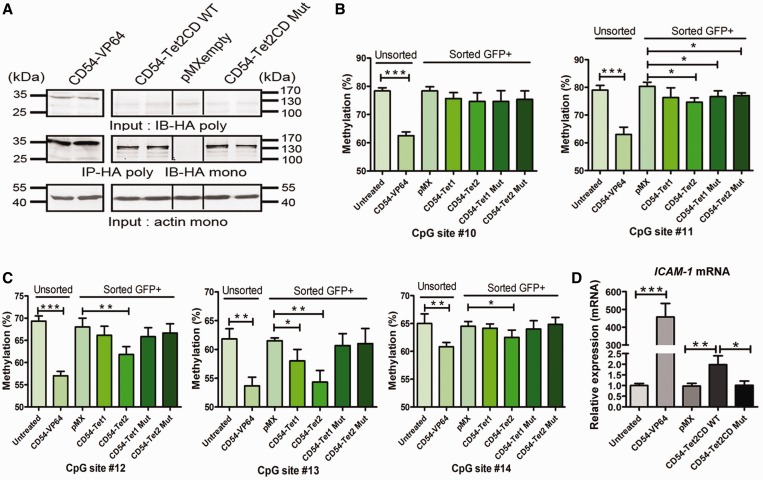
Active DNA demethylation-induced *ICAM-1* gene expression. (**A**) Protein expression of ZF fusion constructs in A2780 host cells. Upper panel: conventional western blot could only detect ZF-VP64; middle panel: HA-tag immunoprecipitation followed by western blot detected ZF-Tet2CD as well as catalytically inactive ZF-TET2CD mutant; lower panel: beta-actin was used as an input control. The results are presented as two biological independent experiments for each ZF-ED fusion construct. (**B**) Quantitative analysis of the methylation levels of target CpG sites in ZF binding region by pyrosequencing after treatment with catalytically inactive CD54-TET1CD and CD54-TET2CD mutant in unsorted and sorted A2780 ovarian cancer cells. (**C**) Quantitative analysis of the methylation levels of target CpG sites in effector domain-targeted region by pyrosequencing after treatment with catalytically inactive CD54-TET1CD and CD54-TET2CD mutant in unsorted and sorted A2780 ovarian cancer cells. (**D**) The analysis of activation of *ICAM-1* gene transcription by qRT-PCR after treatment with catalytically inactive CD54-TET2CD mutant in unsorted and sorted A2780 ovarian cancer cells. Total RNA was isolated, and reverse transcription and qPCR were carried out to assess the expression levels relative to *GAPDH*.

To determine whether the observed demethylation effects were indeed caused by the catalytic activity of the TET enzymes, we constructed catalytic inactive mutants (Figure 2, Supplementary Figure S2A and B and Supplementary Figure S3). Although mutant TET variants were expressed to similar levels compared with their wild-type counterparts (Figure 2A, Supplementary Figure S2C, Supplementary Figure S3B and Supplementary Figure S5C and D), they were severely crippled in inducing genome-wide hydroxymethylation (Supplementary Figure S2D). Upon expressing the different CD54-fusions proteins in a separate set of experiments, again demethylation was observed for CD54-TET2 (and for CD54-TET1 on #13), whereas no demethylation was induced by either TET mutant on the target CpGs (#12–14) (Figure 2C). The observed demethylation for CpG#11 is in line with the effect of the binding of the ZF DNA binding domain (DBD), as also observed for CD54-noED (Figure 1C and 2B).

To investigate whether the TET2-induced active DNA demethylation was able to induce target gene transcription, we investigated *ICAM-1* mRNA levels in treated A2780 cells by quantitative real-time PCR (qRT-PCR). The positive control pMX-CD54-VP64 significantly induced the transcription of *ICAM-1* (457-fold ± 76, *P* < 0.01) (Figure 2D). Interestingly, we also observed a small but significant increase of *ICAM-1* transcription after expression of CD54-TET2 (2.0-fold ± 0.42, *P* < 0.05), but not for CD54-TET2CD mutant (Figure 2D). For CD54-TET1CD or -TET3CD, no expression modulation was detected (Supplementary Figure S4B).

### *EpCAM*-targeted DNA demethylation

**Figure 3.**
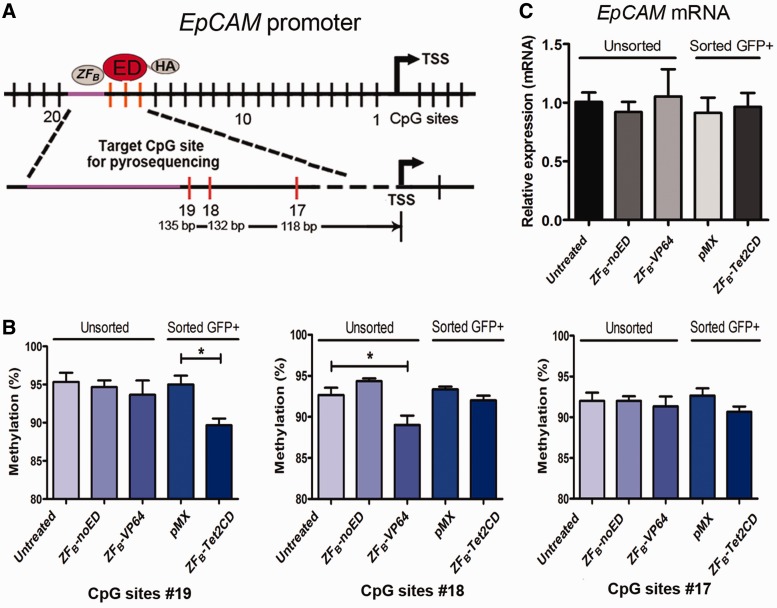
*EpCAM*-targeted DNA demethylation. (**A**) Schematic representation of targeted DNA demethylation in *EpCAM* promoter by epigenetic editing. The binding sites of the ZFs in the promoter of *EpCAM* are depicted, and a magnification of the target region and the actual position of each selected CpG from the transcription start site (target CpG sites are numbered #19, #18 and #17). The purple area represents the ZFP binding site. Gray ovals represent the ZF modules, and the red ovals represent the epigenetic effector domain. (**B**) Quantitative analysis of the methylation levels of CpGs in *EpCAM* promoter by pyrosequencing after treatment with the *EpCAM*-targeted candidate demethylation effector domains in unsorted and sorted A2780 ovarian cancer cells. (**C**) The analysis of activation of *EpCAM* gene transcription by qRT-PCR after treatment with the *EpCAM*-targeted candidate demethylation effector domains in unsorted and sorted A2780 ovarian cancer cells. Total RNA was isolated, and reverse transcription and qPCR were carried out to assess the expression levels relative to *Gapdh*.

Then we set out to check whether targeted TET2CD could also induce demethylation on another target gene. We chose *EpCAM*, which is known to be hypermethylated and silenced in A2780 cells (47). An *EpCAM*-targeting ZF (ZF_B_) (41) was fused to mouse TET2 CD or to the transient activation domain VP64. For three CpG sites located 3′ to the ZF binding region, pyrosequencing primers could be developed (Figure 3A). Significant demethylation was detected in sorted cells expressing ZF_B_-TET2CD for the CpG #19 site (from 93.8 ± 1.7% for pMX to 88.3 ± 3.2% for TET2, *P* < 0.05), which is located directly adjacent to the 3^′^ side of the ZF binding site, compared with pMXempty (Figure 3B). This demethylation was not observed for CpG #17 and #18 (Figure 3B). Interestingly, cells transduced to express ZF_B_-VP64 also showed demethylation, but only for CpG #18 (from 92.6 ± 1.6% for untreated to 88.7 ± 2.3% for ZF_B_-VP64, *P* < 0.05) (Figure 3B), despite a high efficiency of infection (data not shown). In contrast to the data obtained for *ICAM-1*, we did not observe re-activation of *EpCAM* transcription by ZF_B_-TET2 (Figure 3C), but also not for ZF_B_-VP64, which is in line with our previous report (41). So from these data we can conclude that induction of target gene expression might be obtained by targeted TET2, but that the activation of transcription is dependent on the location of the demethylated target CpG sites in the target gene promoter.

### Genome-wide DNA demethylation effects by targeted TET-fusions

**Figure 4.**
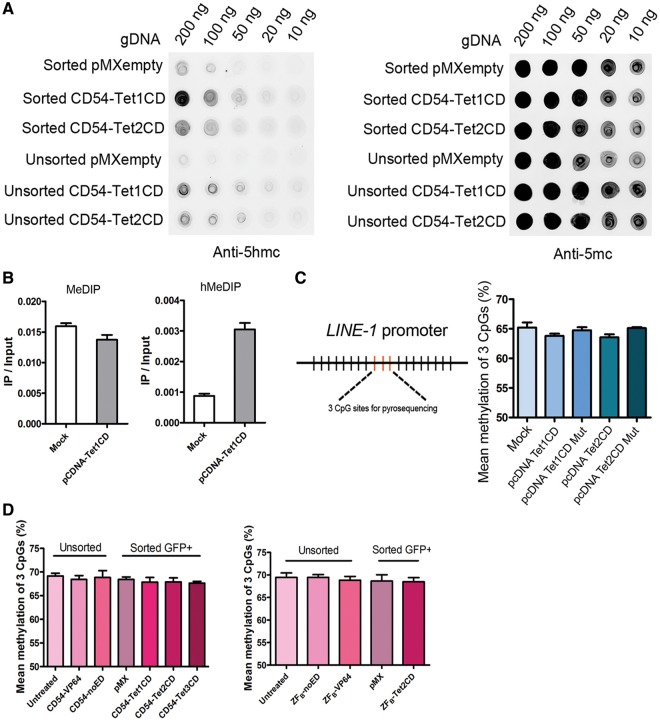
Genome-wide DNA demethylation effects by targeted TET-fusions. (**A**) Fusion of the TET1 and -2 CDs to the *ICAM-1*-targeting DNA binding domains CD54 did result in genome-wide induction of hydroxymethylation. DNA dot-blot assays were performed with genomic DNA isolated from unsorted and sorted A2780 ovarian cancer cells transduced to express pMX-CD54-TET1 or -2CD. (**B**) 5-mC and 5-hmC levels at human long interspersed nuclear element-1 (*LINE-1*) in HEK293T cells transfected with pcDNA-TET1 CD. Quantitative PCR was performed on genomic DNA immunoprecipitated using anti-5-mC antibody (for MeDIP) or anti-5-hmC antibody (for hMeDIP) to evaluate the relative 5-hmC and 5-mC levels (IP/input) at the *LINE-1*. Genomic DNA from HEK293T cells transfected with pcDNAempty serves as a negative control (**C**) Quantitative analysis of the methylation levels of core CpG sites in *LINE-1* promoter by pyrosequencing after treatment with the untargeted candidate demethylation effector domains TET1 and -2CD as well as catalytically inactive TET1 and -2CD mutant in A2780 ovarian cancer cells. (**D**) Quantitative analysis of the methylation levels of core CpG sites in *LINE-1* promoter by pyrosequencing after treatment with the *ICAM-1-* and *EpCAM-*targeted candidate demethylation effector domains in unsorted and sorted A2780 ovarian cancer cells. The results are shown as the mean methylation of three CpG sites.

To address genome-wide effects of our approach, DNA of treated (sorted) cells was analyzed by dot-blot staining: both CD54-TET1CD and CD54-TET2CD could induce genome-wide hydroxymethylation (Figure 4A). To provide some further insights into the extent of hydroxymethylation, *LINE-1* hMeDIP was performed after overexpression of untargeted TET1CD in HEK293 cells. As *LINE-1* sequences are highly repeated human retrotransposon sequences constituting about 17% of the human genome (48), aspecific genome-wide demethylation levels would be directly reflected by lower DNA methylation levels in these repetitive elements. hMeDIP analyses could clearly detect hydroxymethylation in HEK293 cells on *LINE-1* by untargeted TET overexpression (Figure 4B). Despite this seemingly permissiveness of *LINE-1* elements to TET-induced modulation, no actual DNA demethylation could be detected by quantitative pyrosequencing of the three core CpGs in the elements (Figure 4C). Also no DNA demethylation was detected for the three core CpG sites of the *LINE-1* promoter after treatment with either of the targeted candidate effector domains (Figure 4D).

## DISCUSSION

In this study, we induced active DNA demethylation by gene-targeting ZFs fused to TET2, and to a lesser extent by ZF-TET1, but not by ZF-TET3. For *ICAM-1*, the induced loss of DNA methylation in A2780 ovarian cancer cells was associated with a slight increase in gene expression. To our knowledge, this report is the first to actually induce TET-mediated DNA demethylation at a hypermethylated site of interest, and describes an interesting approach for further studying the mechanism of TET-induced DNA demethylation in the endogenous chromatin contexts. Moreover, the approach will open up new avenues to induce sustained re-expression of epigenetically silenced target genes, including tumor suppressor genes.

As already reported by us (43) and others (49), we observed that the ZF-VP64-induced upregulation of gene expression was associated with significant demethylation on the targeted CpG sites in the promoter. Interestingly, here we also report a similar significant DNA demethylation at the ZF binding site (#10, 11) for ZF-only. The observed demethylation at the binding site might reflect inaccessibility of the DNA or competition with Dnmt1, due to steric hindrance by ZF binding, as both the ZF-only and the ZF-VP64 constructs are expressed at high levels. As no DNA demethylation was detected for ZF-only for the other CpGs (#12, 13, 14), and as VP64 is a small domain (7.4 kDa), the VP64-associated demethylation might also be secondary to the re-activation of *ICAM-1* expression. However, bisulfite sequencing revealed that demethylation was limited to the targeted 5 CpGs. In this respect, it is also interesting to note that the TET2-induced DNA demethylation in sorted cells is similar to the VP64-associated demethylation in unsorted cells, despite the fact that the expression induction was only 2.0-fold compared with 457-fold for VP64. Moreover, the induced demethylation at the ZF binding site was less for CD54-TET1CD and CD54-TET2CD compared with CD54-VP64 and ZF-only, reflecting the lower expression level of the large TET-fusions per cell. Importantly, DNA demethylation and/or an effect on gene expression were not observed for the catalytically inactive TET2 mutant. All together our data demonstrated that TET2 and to a lesser extent TET1 induce active DNA demethylation, and that the TET2-induced expression of the gene is not via an indirectly recruited component.

Indeed, apart from the enzymatic activity of the TET family proteins, it was demonstrated that TET proteins might also exert functions independently of their catalytic activity. Helin and colleagues demonstrated that TET1 associates and colocalizes with the Sin3a co-repressor complex in 293T and mouse ES cells (50). Importantly, they observed upregulation of TET1 target genes on TET1 knockdown in DNMT triple-knockout ES cells in which both 5-mC and 5-hmC modifications are absent (50). These results suggest that TET1 might repress gene transcription independent of its catalytic activity. Similarly, two recent studies showed that TET2 recruits O-linked B-*N*-acetylglucosamine (O-GlcNAc) transferase (OGT) (51,52), resulting, e.g. in histone2B O-GlcNAcylation in mouse ES cells (52), which has been reported to positively regulate transcription (53). Besides TET2, TET3 also interacts with OGT, indicating that TET3 might also target OGT to chromatin for gene transcription regulation (51,52). The absence of effect of TET3 in our system could be due to different characteristics of TET3 compared with TET2, including different tissue distribution (10,54,55), as well as catalytic activity (Supplementary Figure S3A) (26). In addition, the cloned CD of TET3CD was larger than the ones for TET1CD and TET2CD; this might explain the even lower expression level of TET3, as a large transgene size might hamper successful production of viral particles as well as efficient integration into the host genome. Moreover, a large effector domain size might also suffer from a decrease in efficiency of accessing the chromatin target site. All together, these considerations suggest that compared with TET1, TET2 exists in different complexes, which might explain our observed differences in gene re-activation between TET1 and TET2, and require further investigations.

In contrast to the data obtained for *ICAM-1*, we observed that targeting of ZF_B_-VP64 led to a significant demethylation only for CpG site #18 in the *EpCAM* promoter, close to the ZF binding site. This inefficient DNA demethylation is in accordance with the lack of induction of gene expression, which might be explained by the higher degree of hypermethylation on target CpG sites of the *EpCAM* promoter versus the *ICAM-1* promoter in these cells. Indeed, also ZF_B_-VP64 failed to induce gene expression in these cells (47). Despite the repressive *EpCAM* chromatin context at this side, TET2 was able to demethylate CpG #19, and this finding has important implications for modulation of genes where single CpGs are known to dramatically affect gene expression, e.g. for *p53* (56).

As the earlier discussed pyrosequencing data might underrepresent the actual effects of the enzymes, we set out to directly detect induced hydroxymethylation. Unfortunately, the T4-Beta-glucosyltransferase assay requires a CCGG site for analysis, which is too far downstream from the current ZF binding site to provide insights. Alternatively, we used hMeDIP to analyze the hydroxymethylation level of the targeted area. Because this method requires the presence of several hydroxymethylated CpG sites in one DNA fragment, it is likely that the efficiency of induced hydroxymethylation is not enough to allow the enrichment of DNA fragment in our study. However, also by using oxidative bisulfite pyrosequencing (44), we could not detect 5-hmC, despite 5-hmC being easily detected in our artificially oxidized hmC control DNA. As this study is the first to interrogate the function of targeted TET at a hypermethylated site, no information is available about the lifetime of 5-hmC within heterochromatin. Also based on its low abundance in most somatic (cancer) cells, it might be likely that 5-hmC is rapidly converted to 5-formylcytosine, 5-carboxylcytosine, e.g. by the targeted TET2 and/or excised by, e.g. TDG.

Currently, many diseases, including cancer, have been associated with epimutations (57,58), and epigenetic marks are being developed as diagnostic or prognostic markers (59–61). Importantly, epigenetic marks are reversible, providing new avenues for therapeutic intervention, and some epigenetic drugs are currently approved for use in the clinic for treatment of hematological malignancies (62–64). To limit associated unwanted aspecific effects, while fully exploiting the reversibility of epimutations, epigenetic writers or erasers can be targeted to specific genes by engineered DNA sequence-specific targeting proteins (38). Using the Nuclear factor kB (NF-kB) DNA binding domain, targeted DNA demethylation was induced by TDG, a T/G mismatch repair enzyme (65), confirming previous studies that TDG plays a role in the DNA demethylation process (28,31,66). In that study, targeted TDG resulted in reduction in methylation levels of 5–10% on the target CpG sites, and an increase in gene expression (65). Together with that study, our study indicates that relatively inefficient DNA demethylation might be sufficient to initiate gene expression re-activation. As observed for, e.g. DNA methylation of *p53*, methylation of just one CpG can be sufficient for silencing (56), suggesting that the location of DNA demethylation is likely important. Towards the goal of specifically targeting a genomic locus, various classes of DBDs can be engineered, such as designer ZF proteins (ZFPs), as used in the current study. Such ZFPs have been fused to transcription activating or repressive domains to form artificial transcription factors, which recruit other proteins to induce (43) or repress (67) the expression of the targeted gene. Fusion of epigenetic writers to ZFPs might provide an approach with potentially more stable gene expression modulation (68–70). Similarly, there are studies reporting on designer Triplex Forming Oligos conjugated with, e.g. DNA methyltransferases (71) and pyrrole-imidazole polyamides conjugated with, e.g. histone deacetylase inhibitors (72).

In other reports, we have employed Epigenetic Editing (the targeted rewriting of epigenetic marks) to achieve downregulation of endogenous genes (68–70). This is the first report where an epigenetic enzyme fused to an engineered DNA binding domain was targeted to an endogenous gene of interest, resulting in upregulation from an epigenetically silenced locus. Although the level of upregulation was low, the approach might be further improved to facilitate endogenous target gene re-expression, while minimizing genome-wide effects. To further increase specificity, other approaches are being investigated, including the split-enzyme approach (73) or by constructing cripple mutants (71). Furthermore, the endogenous gene targeting strategy achieved through Epigenetic Editing is uniquely suited to investigate functions of epigenetic writers and erasers and to elucidate consequences of epigenetic marks at any given chromatin environment, providing insights in gene expression regulation mechanisms.

## SUPPLEMENTARY DATA

Supplementary Data are available at NAR Online.

## FUNDING

Netherlands Organisation for Scientific Research NWO-VIDI [91786373 to M.G.R.]; University Medical Center Groningen (Abel Tasman fellowship to H.C.). Funding for open access charge: The Netherlands Organisation for Scientific Research NWO-VIDI grant number [036.002.446 to M.G.R.].

*Conflict of interest statement*. None declared.

## Supplementary Material

Supplementary Data
