# Supplementary material for: Induced DNA demethylation by targeting Ten-Eleven Translocation 2 to the human ICAM-1 promoter
Source: Nucleic Acids Res. 2013 Nov 3;42(3):1563–74. doi: 10.1093/nar/gkt1019 (PMC3919596; doi:10.1093/nar/gkt1019)
Supplement: Supplementary Data [file supp_42_3_1563__index.html]

Induced DNA demethylation by targeting Ten-Eleven Translocation 2 to the human ICAM-1 promoter — Induced DNA demethylation by targeting Ten-Eleven Translocation 2 to the human ICAM-1 promoter — Supplementary Data 

# Induced DNA demethylation by targeting Ten-Eleven Translocation 2 to the human *ICAM-1* promoter

## Supplementary Data

files

**Files in this Data Supplement:**

- Supplementary Data - pdf file
