# Supplementary material for: Induced DNA demethylation by targeting Ten-Eleven Translocation 2 to the human ICAM-1 promoter
Source: Nucleic Acids Res. 2013 Nov 3;42(3):1563–74. doi: 10.1093/nar/gkt1019 (PMC3919596; doi:10.1093/nar/gkt1019)
Supplement: Supplementary Data [file supp_gkt1019_nar-01769-h-2013-File006.pdf]

Supplementary materials

Supplementary figure 1

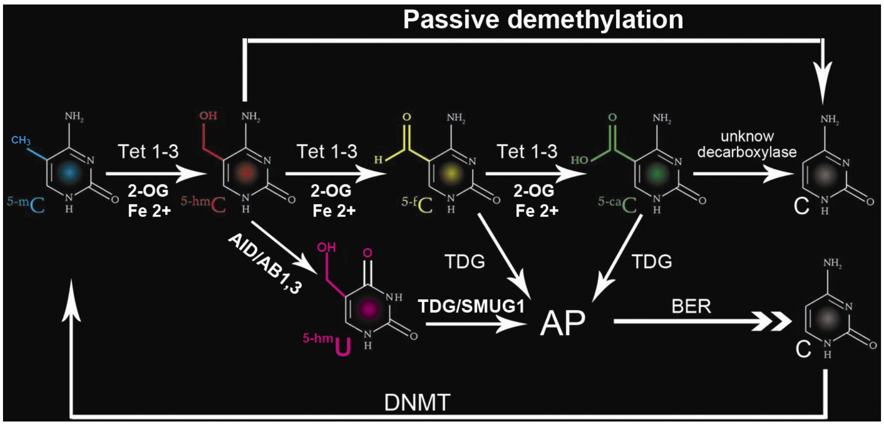

5mC: 5-methylcytosine; 5hmC: 5-hydroxymethylcytosine; 5fC: 5-formylcytosine; 5caC: 5-carboxylcytosine; 5hmU: 5-hydroxymethyluracil; AP: abasic site; C: cytosine;

TET: ten eleven translocation; AID: activation-induced deaminase;

APOBEC: apolipoprotein mRNA editing complex; TDG: thymine DNA glycosylase; SMUG: single-strand-selective monofunctional uracil-DNA glycosylase; BER: base excision repair pathway;

Supplementary figure 2

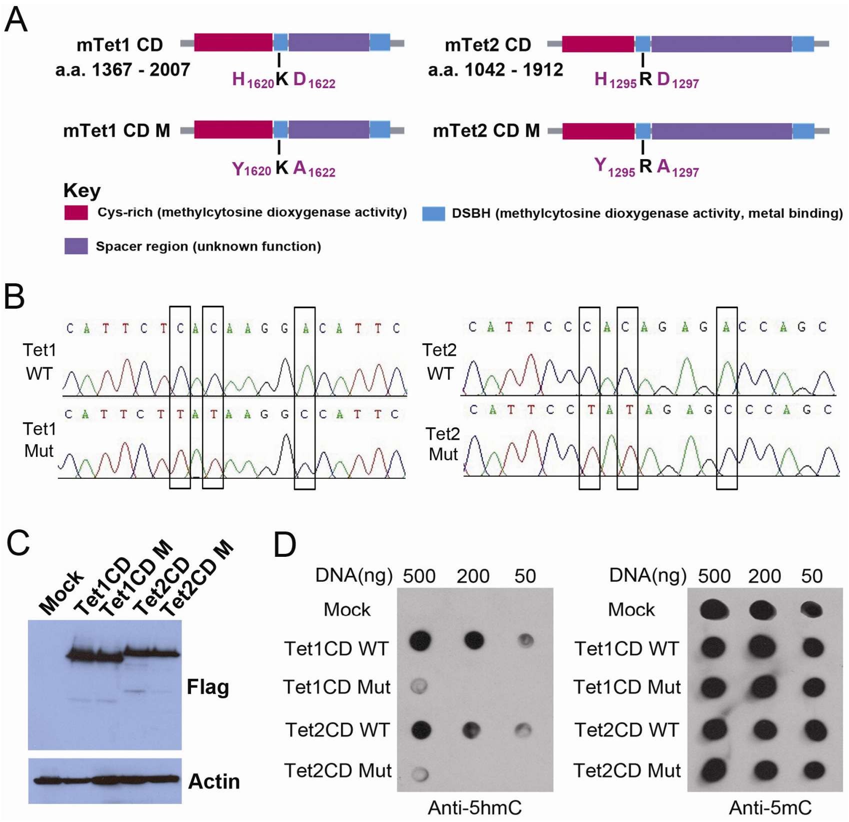

Supplementary figure 3

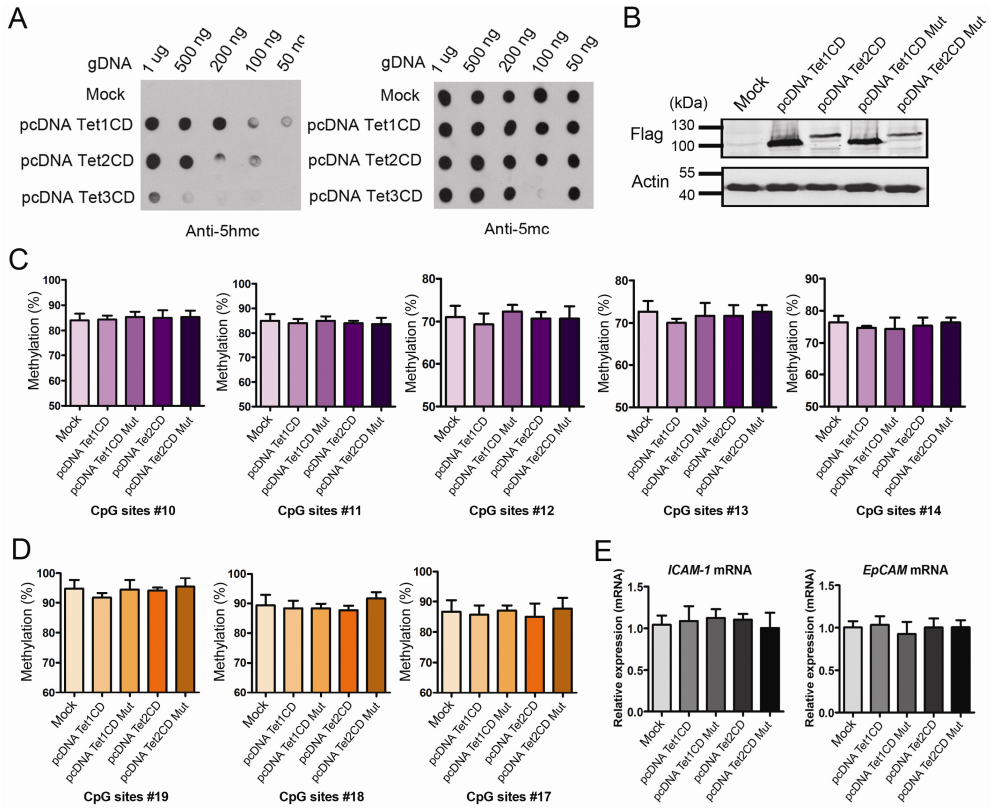

Supplementary figure 4

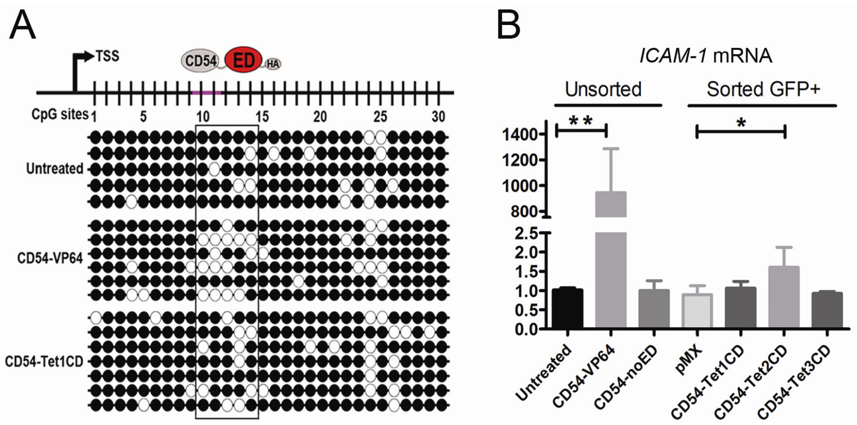

Supplementary figure 5

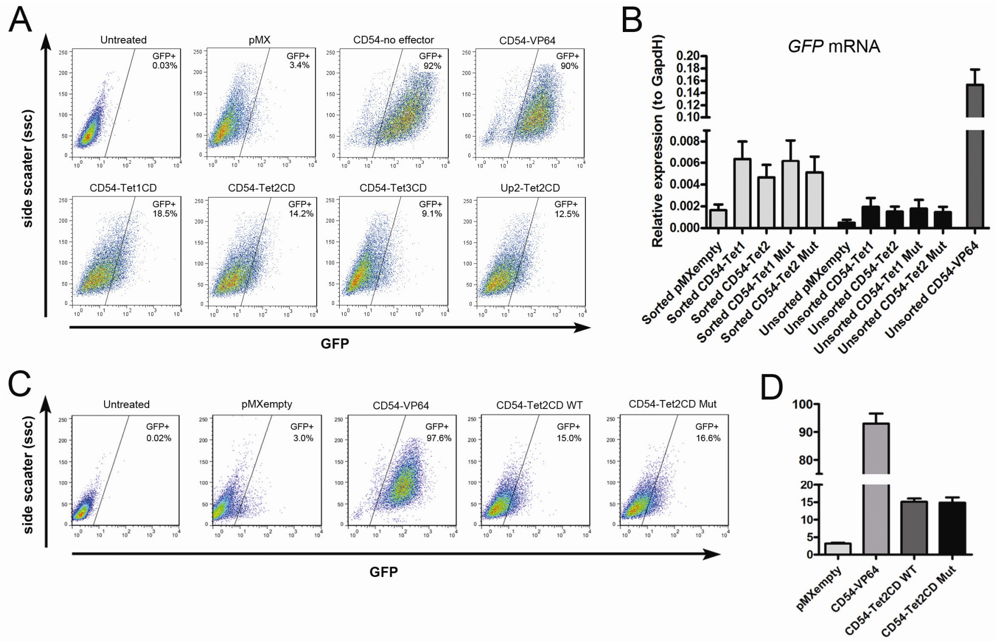

Supplementary figure 6

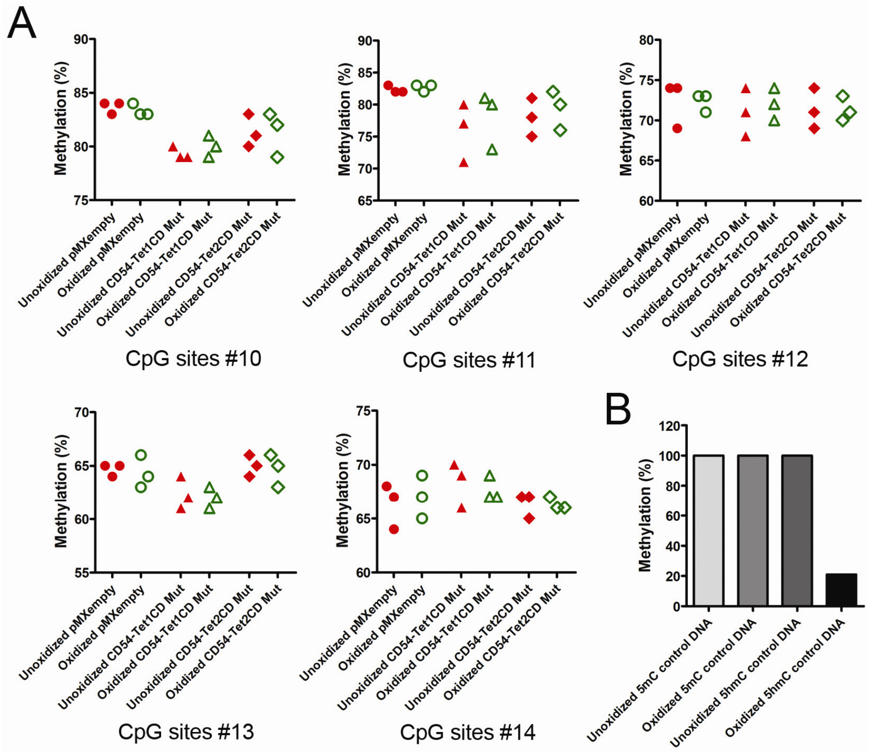

Supplementary figure legends

Supplementary figure 1 Proposed models of TET-dioxygenase initiated DNA demethylation pathways within targeting demethylation process.

Supplementary figure 2 The mTet1 and mTet2 catalytic domain mutants and functional verification. (A) Schematic diagram of wild type mouse Tet1,Tet2 (mTet1CD and mTet2CD) and catalytically inactive mutant Tet1, Tet2 (mTet1CD- M and mTet2CD-M) proteins. (B) mTet1 and mTet2 catalytic domain mutants verified by DNA sequencing. (C) Protein expression of flag-tagged Tet1CD or Tet2CD and mutants by western blot, beta-actin was used as a loading control. (D) Dot blot data show global 5-hmC levels in HEK cells after transfection with pcDNA-Tet1CD or Tet2CD and Tet1CD or Tet2CD mutants, 5mC was used as a loading control.

Supplementary figure 3 Overexpression untargeted TET did not affect methylation status of *ICAM-1* and *EpCAM* promoter as well as transcription levels. (A) 5- Hydroxymethylcytosine levels increased genome-wide after expression of untargeted TET1, -2 or -3 catalytic domains. DNA dot-blot assays were performed with genomic DNA isolated from transfected A2780 ovarian cancer cells. (B) Protein expression of untargeted flag-tagged TET1, -2CD and mutants by western blot in transfected A2780 ovarian cancer cells, beta-actin was used as a loading control. (C) Quantitative analysis of the methylation levels of target CpG sites in *ICAM-1* promoter by pyrosequencing after treatment with the untargeted candidate demethylation effector domains TET1, -2CD and mutants in transfected A2780 ovarian cancer cells. (D) Quantitative analysis of the methylation levels of target CpG sites in *EpCAM* promoter by pyrosequencing after treatment with the untargeted candidate demethylation effector domains TET1, -2CD and mutants in transfected A2780 ovarian cancer cells. (E) The analysis of activation of *ICAM-1* and *EpCAM* gene transcription by qRT-PCR after treatment with the untargeted candidate demethylation effector domains TET1, -2CD and mutants in transfected A2780 ovarian cancer cells. Total RNA was isolated, and reverse transcription and qPCR were carried out to assess the expression levels relative to Gapdh.

Supplementary figure 4 Determine the target CpG sites in the *ICAM-1* promoter by bisulfite sequencing as well as detection of *ICAM-1* transcription in A2780 ovarian cancer cells transduced to express all *ICAM-1*-target constructs. (A) Methylation status of target CpG sites in the *ICAM-1* promoter determined by bisulfite sequencing after treatment with CD54 fused to the transcription activator VP64 or epigenetic effector domains TET1CD. Schematic representation of CpG's in the *ICAM-1* promoter in the top of the panel, the first CpG located downstream of the transcription start site is represented as number 1. Gray and red ovals represent the *ICAM1*-targeting zinc-finger (CD54) and the epigenetic effector domain, respectively. The purple area represents the CD54 zinc-finger binding sites in the *ICAM-1* promoter. The black dots indicate methylated C's and the white dots indicate unmethylated C's. The vertical rectangular box includes five CpG sites which represent the selected target CpG sites for pyrosequencing. (B) The analysis of activation of *ICAM-1* gene transcription by qRT-PCR after treatment with the targeted candidate demethylation effector domains TET1, -2,-3CD in unsorted and sorted A2780 ovarian cancer cells.

Supplementary figure 5 pMX-ZF-ED-IRES-GFP fusion constructs was highly expressed in sorted GFP+ than unsorted A2780 cells as well as targeted TET2CD and TET2CD mutant have the same transduction efficiency. (A) Dot plots of FACS analysis of GFP expression to evaluate the transduction efficiency in A2780 ovarian cancer cells transducted with all of pMX-ZF-ED-IRES-GFP constructs. (B) The analysis of expression levels of *GFP* transcription by qRT-PCR after treatment with the *ICAM-1*-targeted candidate demethylation effector domains CD54-TET1, -2CD as well as mutants in unsorted and sorted A2780 ovarian cancer cells. Total RNA was isolated, and reverse transcription and qPCR were carried out to assess the expression levels relative to Gapdh. (C) Dot plots of FACS analysis of GFP expression to evaluate the transduction efficiency in A2780 ovarian cancer cells transducted with CD54-TET2CD and CD54-TET2CD mutants. (D) Three biological independent experiments of FACS analysis of GFP expression in A2780 ovarian cancer cells transducted with CD54-TET2CD and CD54-TET2CD mutants.

Supplementary figure 6 (A) Quantitative sequencing analysis of methylation and hydroxymethylation levels of target CpG sites at single-base resolution by combining oxidative bisulfite treatment and pyrosequencing in sorted A2780 ovarian cancer cells transduced to express CD54-TET1 mutants and CD54-TET2CD mutants. (B) Evaluate oxidation efficiency and experimental methods by using synthetic 5mC and 5hmC control double-stranded DNA.

Supplementary table 1 PCR and sequsncing primer

Primer name	Sequence (5'- 3')	Application
pMX-Tet1 F	AATACGCGTGAAGCTGCACCCTGTGACTG	Clone Tet1 catalytic domain into the pMX vector
pMX-Tet1 R	GTTAATTAAGACCCAACGATTGTAGGGTCCC	
pMX-Tet2 F	CGACGCGTCAAAGTCAGAATGGCAAATG	Clone Tet2 catalytic domain into the pMX vector
pMX-Tet2 R	CCTTAATTAATACAAATGTGTTGTAAGGCCC	
pMX-Tet3 F	GATACGCGTGAGTTCCCTACCTGCGATTG	Clone Tet3 catalytic domain into the pMX vector
pMX-Tet3 R	GTTAATTAAGATCCAGCGGCTGTAGGGGC	
GAPDH-F	CCACATCGCTCAGACACCAT	qRT-PCR for *Gapdh*
GAPDH-R	GCGCCCAATACGACCAAAT	
GAPDH-Probe	CGTTGACTCCGACCTTCACCTTCCC	
GFP-F	ACGTAAACGGCCACAAGTTC	qRT-PCR for *GFP*
GFP-R	AAGTCGTGCTGCTTCATGTG	
ICAM-1 BS-F1	TAAGTTGGAGAGGGAGGATTTGAG	Nested PCR for *ICAM-1* bisulphite-sequencing
ICAM-1 BS-F2	GATTTAAGTTTAGTTTGG	
ICAM-1 BS-R1	CTATCTCTAACCCCTCCTTCCCAT	
ICAM-1 BS-R2	TCACCTAAAAACAAAACCCC	
ICAM-1 Pyro-F	GGGGAAGTTGGTAGTATTTAAAAGT	PCR and sequencing for *ICAM-1* pyrosequencing
ICAM-1 Pyro-R	CCTTCCCCTCCCAAACAAATACTACAATTA	
ICAM-1 Pyro-seq	ATTTCCCAACTAACAAAATACCC	
EpCAM Pyro-F	TGGGGGAGGGGAGTTTATT	PCR and sequencing for *EpCAM* pyrosequencing
EpCAM Pyro-R	ACCCAACTCCACAACTCT	
EpCAM Pyro-seq	AGGGGAGTTTATTTATTTTTTTA	
LINE-1 Pyro-F	TTTTGAGTTAGGTGTGGGATATA	PCR and sequencing for *LINE-1* pyrosequencing
LINE-1 Pyro-R	AAAATCAAAAAATTCCCTTTC	
LINE-1 Pyro-seq	AGTTAGGTGTGGGATATAGT	
ICAM-1 hMe-F	AGACCGTGATTCAAGCTTAGCCTG	MeDIP/hMeDIP-qPCR for *ICAM-1* promoter
ICAM-1 hMe-R	AGTTATTTCCGGACTGACAGGGT	
LINE-1 hMe-F	CCGAAGCAGGGCGAGGCATTG	MeDIP/hMeDIP-qPCR for *5' LINE-1*
LINE-1 hMe-R	ATCAGCGAGATTCCGTGGGCG	
5hmC DNA-F	AGTGAAGTTGGTAGATTGAGTTAG	PCR and sequencing for hmC/mC control DNA pyrosequencing
5hmC DNA-R	TAAACAACCCTTACTCTCCTACAAAATT	
5hmC DNA-seq	GGTAGATTGAGTTAGGT	

Supplementary table 2 5mC and 5hmC control dsDNA sequence

Name	Sequence (5'- 3')
5mC control DNA	CAGTGAAGTTGGCAGACTGAGCCAGGTCCCACAGATGCAGTGAC(**m**)**CG**GAGTCATTGCCAAACTCTGCAGGAGAGCAAGGGCTGTCTATAGGTGGCAAGTCA
5hmC control DNA	CAGTGAAGTTGGCAGACTGAGCCAGGTCCCACAGATGCAGTGAC(**hm**)**CG**GAGTCATTGCCAAACTCTGCAGGAGAGCAAGGGCTGTCTATAGGTGGCAAGTCA
